# Extensive range contraction predicted under climate warming for two endangered mountaintop frogs from the rainforests of subtropical Australia

**DOI:** 10.1038/s41598-022-24551-5

**Published:** 2022-11-23

**Authors:** Liam Bolitho, David Newell

**Affiliations:** Faculty of Science and Engineering, Southern Cross University, PO Box 157, Lismore, NSW 2480 Australia

**Keywords:** Climate-change ecology, Ecological modelling

## Abstract

Montane ecosystems cover approximately 20% of the Earth's terrestrial surface and are centres of endemism. Globally, anthropogenic climate change is driving population declines and local extinctions across multiple montane taxa, including amphibians. We applied the maximum entropy approach to predict the impacts of climate change on the distribution of two poorly known amphibian species (*Philoria kundagungan* and *Philoria richmondensis*) endemic to the subtropical uplands of the Gondwana Rainforests of Australia, World Heritage Area (GRAWHA). Firstly, under current climate conditions and also future (2055) low and high warming scenarios. We validated current distribution models against models developed using presence-absence field data. Our models were highly concordant with known distributions and predicted the current distribution of *P. kundagungan* to contract by 64% under the low warming scenario and by 91% under the high warming scenario and that *P. richmondensis* would contract by 50% and 85%, respectively. With large areas of habitat already impacted by wildfires, conservation efforts for both these species need to be initiated urgently. We propose several options*,* including establishing ex-situ insurance populations increasing the long-term viability of both species in the wild through conservation translocations.

## Introduction

Mountain ecosystems cover approximately 20% of the Earth's terrestrial surface^1^ and are home to an exceptional concentration of endemic taxa that typically have small isolated ranges, narrow climatic niches, and are inherently reliant on the moist, comparatively cooler environments^2–4^. As temperature increases are brought about through climate change, upslope movement of montane taxa will be required in order to maintain thermal preferences^5^. However, this will be particularly problematic when there are barriers to dispersal, or for species with poor dispersal ability^6^ and those that are already occupying mountain tops. They are effectively stranded within a climatic niche that is shifting^7^. Consequently, climate change poses a severe threat to montane communities, with shifts in distribution and local extinctions associated with a changing climate already documented across multiple taxa, including plants, fungi, mammals, birds and amphibians^8–12^. This is particularly evident in tropical taxa and there have been relatively few assessments undertaken in subtropical species and in landscapes that are less scarped.

Although Australia is considered the world's flattest continent^13^, its montane regions contain highly diverse communities of endemic taxa^14–16^. Notable among these regions is the Gondwana Rainforests of Australia, World Heritage Area (GRAWHA), in subtropical eastern Australia, which is home to rare and threatened taxa, many of which are restricted largely or entirely to these forests^17–20^. Despite the high levels of protection afforded by World Heritage listing, climate change is a key threat to the GRAWHA^19^. This was made evident in 2019–2020, when unprecedented wildfires burnt approximately 53% of the GRAWHA^21^ during one of the hottest and driest Australian summers on record^22,23^. The average temperature of this region has already warmed by more than one-degree Celsius since 1910^24^, with recent models predicting this will climb to 1.4–2.1 °C of warming by 2050 above the 1961–1990 baseline^25^. In addition, forecasted increases to rainfall variability and reduced moisture availability from cloud-stripping due to a shifting cloud base height^19,26^ will also lead to deeper and more frequent droughts in this region^19,26^.

Available moisture is explicitly linked to breeding in amphibians^27,28^ and as ectotherms, amphibians rely on environmental temperatures^27,28^. Due to these characteristics, amphibians are expected to be particularly susceptible to climate change^27–30^. For the 40 + amphibian species occupying montane rainforest habitats in the GRAWHA^17^, the increasing pressures of climate change are likely to lead to substantial range contractions and local extinctions for some^31,32^. Identifying the most at-risk species is crucial if we are to maintain the full suite of amphibian diversity.

Species Distribution Modelling (SDM) is a powerful method for predicting where a species occurs in the landscape and how a species distribution will respond to changing climatic conditions^33–35^. This approach uses the relationship between species' occurrences and climatic variables at those locations, such as average annual rainfall and average maximum temperature in the warmest month, to estimate its geographic distribution^36,37^. The use of SDM's to predict changes to a species' geographic range in response to future climate change scenarios has increased substantially over the last decade as the method has been refined^38–40^. This is a valuable tool in biodiversity conservation as it provides insight into conservation actions to avoid or minimise future declines or extinctions of threatened species^38–41^.

Among the amphibian species found within the GRAWHA are six of the seven described species of Mountain frog (*Philoria*). These allopatric species occur as scattered mountaintop endemics^42^ and primarily occupy headwater streams and seepages within rainforest or adjoining wet sclerophyll vegetation communities^43,44^. Males call from nest burrows constructed in saturated soil, often associated with boggy stream margins. Metamorphosis occurs within these nests, which require constant moisture^42^. *Philoria kundagungan* and *P. richmondensis* are thought to be among the most threatened of these six species, with both listed as Endangered under the Australian Commonwealth's *Environment Protection and Biodiversity Conservation Act 1999*. The restriction of *P. kundagungan* and *P. richmondensis* to rainforest and adjoining wet forest, and the specific reliance of these frogs on moist or saturated microhabitats, suggest that they may be particularly sensitive to increasing temperatures and rainfall variability predicted under future climate change scenarios^20^. The geographic distribution of both species was recently modelled in order to produce high-resolution mapping outputs of their current distributions^45,46^, however, the approach used could not predict changes to a species' geographic range in response to future climate change scenarios. These recent estimates gave us an opportunity to further validate our species distribution model and in turn, our estimates of the impact of projected climate change on their future distribution. This study sought to assess the impact of future climate change on the distribution of these species by developing an SDM for both species under current climate conditions and under future (2050) low (1 °C increase) and high (2–3 °C increase) warming scenarios and to make specific recommendations for conservation action on the basis of the results.

## Methods

### Species data and study area

Occurrence records were sourced from the New South Wales Office of Environment & Heritage and Queensland Parks and Wildlife Service; however, few records were available from these datasets within the date range of the current bioclimatic variables used in this study (1976–2005). *Philoria richmondensis* was only described in 2004 and only six localities existed for *P. kundagungan*^43^. Due to this limitation, records for *P. kundagungan* and *P. richmondensis* were also obtained from survey data collected by Bolitho et al.^45^ and Heard et al.^46^. Occurrence records were converted to a 1-km resolution raster dataset to align with the bioclimatic data used in the SDM. After duplicate grid cells were removed, 34 1-sq. km grid cells contained occurrence records for *P. kundagungan* and 28 for *P. richmondensis*. We acknowledge that some warming has occurred since the date range of the current environmental data used in this study however, no local extinctions are known to have occurred in *P. richmondensis* and one is suspected for *P. kundagungan*. Study areas were defined for both species as the minimum convex polygon of occurrence records plus a 100 km buffer to include adjacent high-elevation areas. The study area of each species bounded all spatial data used and generated by our SDMs.

### Environmental data

Fourteen current and future bioclimatic variables (B01, B05-6, B08-14, B16-19) were sourced from the 'All future layers for Australia' data set^47^, at 1-km resolution and clipped to the extent of our study areas. We selected these climatic variables as they have previously been shown to play important roles in the performance of SDM's of two endemic ectotherms inhabiting the upland cloud forests of the GRAWHA^48^. Current bioclimatic variables were generated from aggregating monthly data from 1976 to 2005 from the Australia Water Availability Project using WorldClim methodology^49^. Future climate variables were projected to the year 2055 using the CSIRO-MK3 General Circulation Model under two representative concentration pathways (RCPs); RCP 4.5 which predicts a 1.6–2.5 °C increase in the average global temperature in the medium term (2041–2060) and RCP 8.5, which represents the worst-case climate change scenario and predicts a 1.9–3.0 °C increase in the average global temperature in the medium term (2041–2060)^25^. A digital elevation model using a 1-km grid was sourced from Geoscience Australia^50^ and was included as an environmental variable in all current and future species distribution models.

### Modelling and analysis

Species distribution models were predicted using the maximum entropy model (MaxEnt)^37^ in the Biomod2 R package^51^. The MaxEnt model implements a maximum entropy algorithm to calculate the current geographic range of a species' climatic niche from species occurrence data, pseudoabsences and a set of baseline climate and environmental data grids^37^. Once a model had been constructed for current climatic conditions, future climate data generated from global circulation models can be included in a MaxEnt projection model to identify areas of range contraction or expansion under future climate scenarios^37^. All modelling in this study was based on climatic and elevation data and ignored other factors contributing to a species' full ecological niche, such as biotic interactions.

The mean of ten randomly seeded, cross-validated, duplicate models was used to obtain all model outputs and evaluations. All current and future models included the complete set of bioclimatic variables (B01, B05-6, B08-14, B16-19) and elevation as MaxEnt accounts for redundant variables, such as highly correlated variables^52^. All models were run with 5000 randomly generated pseudoabsences, confined to the study area, and all other parameters were set to default.

For each species, the Maxent model was used to calculate the probability of each 1-km grid cell in the study area encompassing the species' climatic niche under current climatic conditions. Future climate data for the year 2055 under two RCPs (RCP 4.5 and RCP 8.5) were then added to the model to calculate the probability of each 1-km grid cell in the study area encompassing the species' climatic niche under future climatic conditions. Raster outputs of the probability of occurrence from final models were converted into binary values; climatically suitable and climatically unsuitable habitat using the mean optimised true skill statistic cutoff value. This value generates a geographical climatic niche range that maximises the combined rate of correctly predicted presences and absences. All species distribution models were performed in RStudio (version 1.4; Rstudio Team 2020) using the Biomod2 package^51^. All *Philoria* species are allopatric and genetic data shows they have speciated based on millions of years of isolation^43,44^, as such^43^, climatically suitable areas for *P. kundagungan* and *P. richmondensis* predicted within the known range of a neighbouring *Philoria* species were removed. Changes in the area of climatically suitable habitat between current and future climates were analysed in ArcMap 10.4.1 using the calculate geometry tool. Model performance was assessed using the Area Under the Curve of the receiver operating characteristic (AUC). AUC values of 0.8–0.9 represent a good fit between model and test data, while values over 0.9 denote an excellent fit^53^.

To further validate our species distribution models (SDMs), we compared current species distribution models (SDMs) generated by this study with a *P. kundagungan* SDM developed by Bolitho et al.^45^ and a *P. richmondensis* SDM developed by Heard et al.^46^. These were based on presence-absence field data collected across the full range of each species using a patch occupancy survey approach^54^. As these earlier SDMs encompass a broad range of occupancy probabilities (0.05–1) and include low-quality *Philoria* habitats, we also compared a subclass of occupancy probabilities ranging from 0.5 to 1.0, representing core *Philoria* habitat. AUC could not be compared as it was not reported in these studies.

## Results

### Current distribution

#### Philoria kundagungan

**Figure 1:**
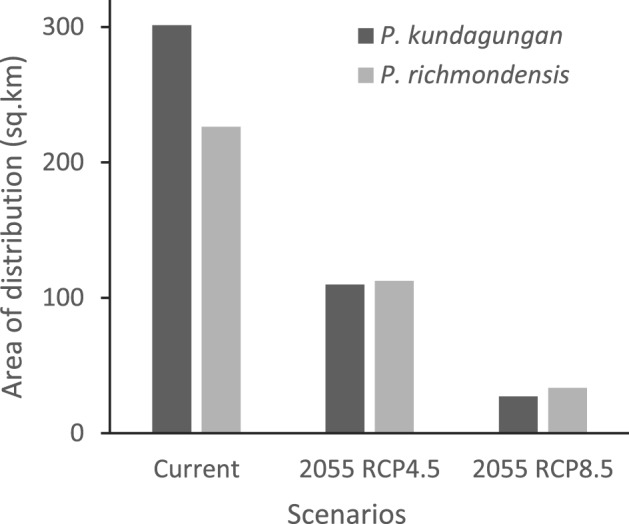
Area (sq. km) of the predicted distribution of *P. kundagungan* and *P. richmondensis* under current and future climate scenarios.

The performance of the current habitat model for *P. kundagungan* was excellent with a mean AUC value of 0.995. This model identified 73.2% of the distribution and 83.1% of the core habitat that was modelled by Bolitho et al.^45^. The mean temperature of the wettest quarter (BIO_08) had the strongest influence on *P. kundagungan* distribution followed by minimum temperature of the coldest month (BIO_06) and then mean temperature of the coldest quarter (BIO_11); (Table 1). The mean optimised true skill statistic cut-off value for the model was 0.317. The current bioclimatic envelope encompassed all known occurrence records and covered an estimated area of 301.2 sq. km (Fig. 1).

**Table 1:** Variables used in modelling current and future distributions of *P. kundagungan* and *P. richmondensis* and their permutation importance (%).

Bioclimatic variable	*P. kundagungan* permutation importance (%)	*P. richmondensis* permutation importance (%)
BIO1—annual mean temperature	0.0	0.0
BIO5—max temperature of warmest month	0.0	0.0
BIO6—min temperature of coldest month	26.5	26.4
BIO8—mean temperature of wettest quarter	32.6	31.9
BIO9—mean temperature of driest quarter	0.0	0.0
BIO10—mean temperature of warmest quarter	0.0	0.1
BIO11—mean temperature of coldest quarter	21.5	0.0
BIO12—annual precipitation	0.0	0.6
BIO13—precipitation of wettest month	11.2	3.1
BIO14—precipitation of driest month	0.0	0.0
BIO16—precipitation of wettest quarter	3.5	0.0
BIO17—precipitation of driest quarter	0.0	19.0
BIO18—precipitation of warmest quarter	1.9	11.8
BIO19—precipitation of coldest quarter	1.6	0.0
elevation	1.2	7.0

#### Philoria richmondensis

The performance of the current habitat model for *P. richmondensis* was excellent with a mean AUC value of 0.986. This model identified 38.5% of the distribution and 87.4% of the core habitat that was modelled by Heard et al.^46^. The mean temperature of the wettest quarter (BIO_08) had the most substantial influence on *P. richmondensis* distribution, followed by the minimum temperature of the coldest month (BIO_06) and then precipitation of the driest quarter (BIO_17); (Table 1). The mean optimised true skill statistic cut-off value for the model was 0.214. The current bioclimatic envelope encompassed all known occurrence records and covered an estimated area of 226.4 sq. km (Fig. 1).

### Future predicted distribution

**Figure 2:**
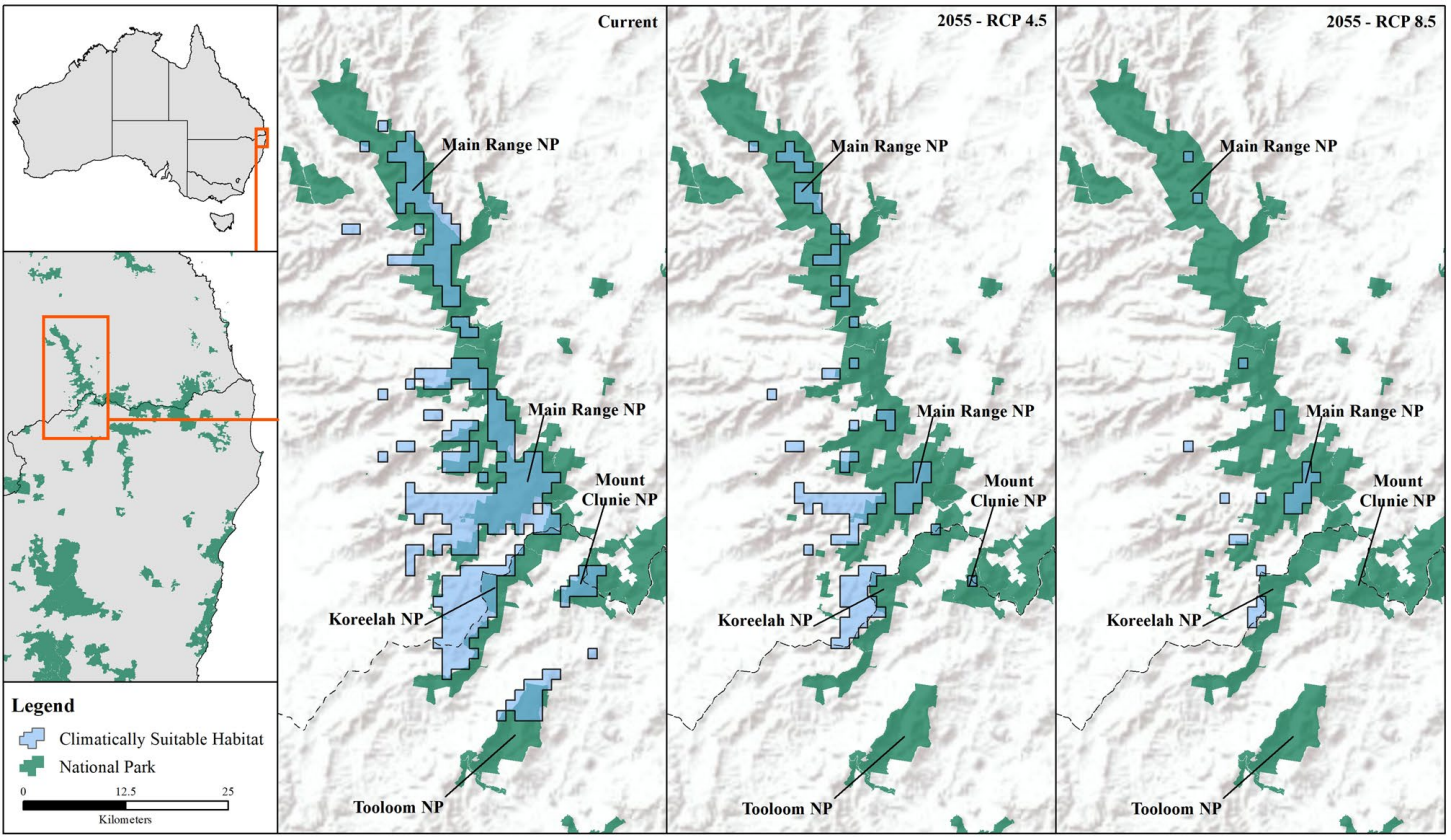
Predicted distribution of *P. kundagungan* under current conditions and, RCP4.5 and RCP8.5 climate scenarios in 2055. This figure was produced using ArcMap 10.4.1, https://www.esri.com/en-us/home.

**Figure 3:**
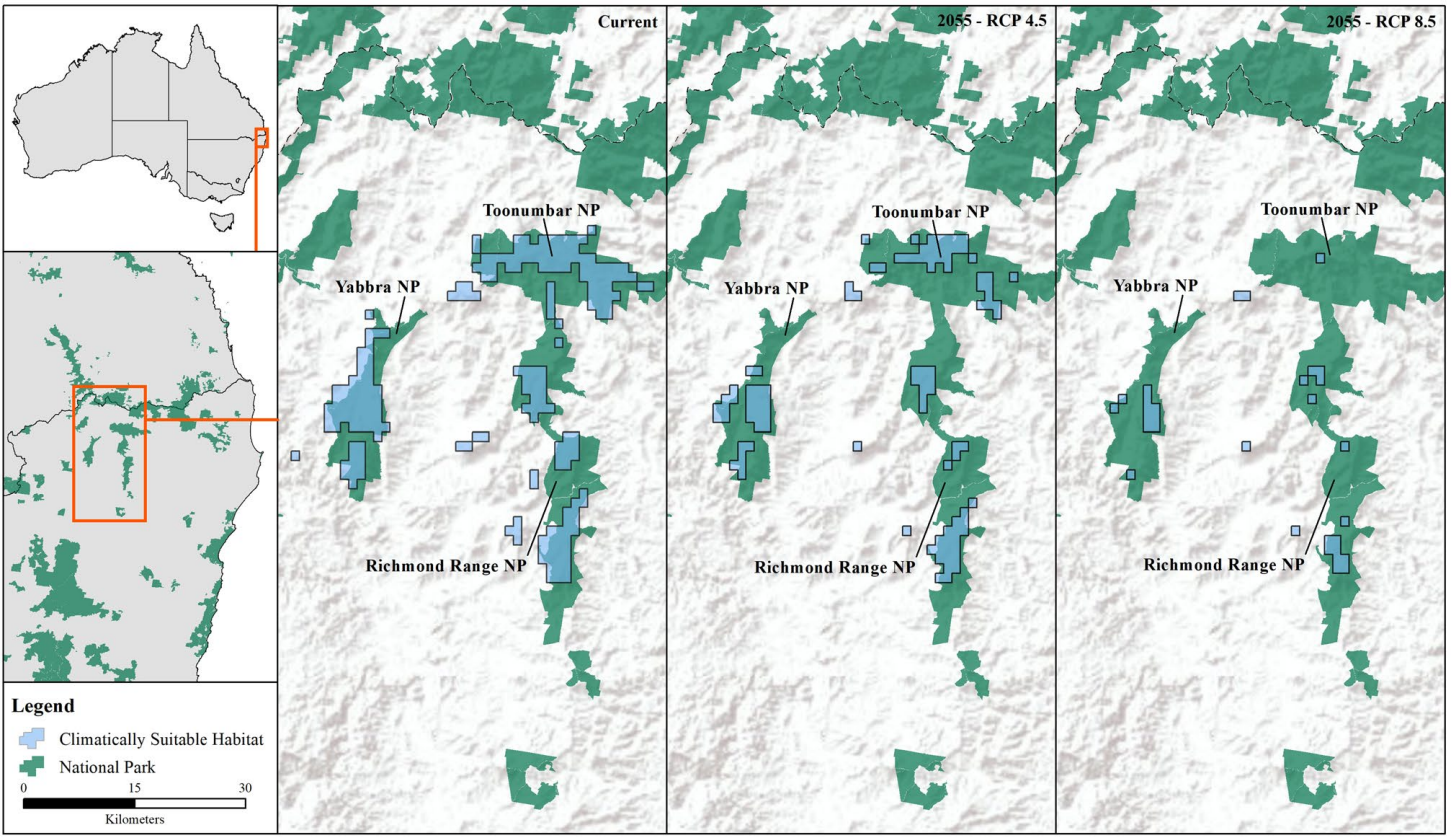
Predicted distribution of *P. richmondensis* under current conditions and, RCP4.5 and RCP8.5 climate scenarios in 2055. This figure was produced using ArcMap 10.4.1, https://www.esri.com/en-us/home.

All climate-warming scenarios predicted substantial decreases in the distribution of both *Philoria* species (Figs. 2, 3). The current *P. kundagungan* distribution is predicted to contract by 191.4 sq. km (64%) under the RCP 4.5 scenario and by 274.0 sq. km (91%) under the RCP 8.5 scenario (Figs. 1, 3). The current *P. richmondensis* distribution is predicted to contract by 113.7 sq. km (50%) under the RCP 4.5 scenario and by 192.8 sq. km (85%) under the RCP 8.5 scenario (Figs. 1, 2). For both species, we did not find any areas of suitable habitat under future climates not currently encompassed by the species' present range.

## Discussion

This study has shown that the distribution of *P. kundagungan* and *P. richmondensis* can be predicted from a set of bioclimatic variables and elevation. Our future climate modelling predicts a substantial contraction of climatically suitable habitat for both species, exposing populations to novel climatic conditions by the year 2055 under both high and low warming scenarios. Without extensive changes to current and stated policies, it is plausible that the climate could track the high warming scenario (RCP8.5)^55^, causing extreme weather events likely to push environmental conditions outside of the environmental tolerance limits of many species in this region before 2050^56^.

### Model performance

Uncertainty in distribution modelling is unavoidable in this study as modelling was based only on climatic and elevation data, and ignored other factors contributing to each species' full ecological niche, such as vegetation type. However, our SDMs for current distribution covered all known populations of *P. kundagungan* and *P. richmondensis* and were similar to core habitat identified in previous studies^45,46^, which were based on presence-absence, vegetation and elevation data, and did not include bioclimatic variables generated from aggregating three decades of monthly climatic data used in this study. This broad consensus with previous studies and our models' high AUC model scores suggests this study accurately identifies the current distribution of *P. kundagungan* and *P. richmondensis*.

### Species distribution

The distribution of *P. kundagungan* and *P. richmondensis* are currently restricted to only a few narrow bands of mountainous habitat, however, even under the lower warming scenario, this study predicts both species will suffer large range contractions (> 50%) by 2055. Our prediction of current distributions may already include a contraction as most occurrence data is from recent surveys and the regional l climate has already warmed by over 1 °C since 1960^24^. Indeed, Bolitho et al.^45^ identified several lower elevation locations for which *P. kundagungan* has already seemingly vanished within the last 20 years, despite concentrated search efforts. By 2055, under a high warming scenario, both species are predicted to lose more than 85% of their habitat, with only the highest elevations in the landscape providing suitable climatic refuge. Under both warming scenarios, suitable habitat is predicted to disappear at lower elevations exposing current populations of both species occupying the lower elevations of their range to novel climatic conditions by 2055. Similar range shifts are frequently predicted for amphibians occupying montane environments in tropical and sub-tropical habitats^57–59^. Our results are consistent with previous studies advising that climate change is likely to threaten *P. kundagungan* and *P. richmondensis*^31,32^, a threat common to most amphibians occupying montane habitats in eastern Australia^20,31,32,60^.

### Mechanism driving the contraction of climatically suitable habitat

Amphibian species exposed to novel climatic conditions are expected to experience multiple impacts, such as shifts in breeding phenology, increased susceptibility to disease, and decreasing body condition^22,27,28^. However, a reduction in breeding activity caused by more frequent and severe droughts is likely to become a key mechanism driving the contraction of climatically suitable habitat in *P. kundagungan* and *P. richmondensis,* an alarming process already documented in other Australian frog species^61,62^. Large reductions in breeding activity have been observed in *P. kundagungan* and *P. richmondensis* due to rainfall deficits in the previous winter and early spring^46^. With the frequency and severity of drought projected to increase over the coming decades, breeding success in *P. kundagungan* and *P. richmondensis* is likely to decline in areas of the habitat more prone to drying, leading to population declines, range contractions and local extinctions.

Habitat degradation caused by increasingly frequent extreme wildfires is also likely to become a key mechanism driving the contraction of climatically suitable habitat for many species^63^, including *P. kundagungan* and *P. richmondensis*. Both species are dependent on the cool, wet microhabitats found in rainforests^43,45^. However, drier conditions paired with increasing temperatures have increased the annual average area of forest burnt by wildfires in Australia by 350% since 1994 (1988–2001 average) and by 800% when including fires in 2019^64^ and wildfires are now beginning to penetrate ecosystems never previously known to burn^65,66^. Indeed, extreme wildfires in 2019/2020 burnt approximately 30% of *P. kundagungan* habitat and 12% of *P. richmondensis* habitat, which likely caused direct frog mortalities while severely degrading sensitive breeding habitat^46^. Sub-tropical rainforests can take many decades to recover from severe wildfire^67^ and are susceptible to regeneration failure^68^. Moreover, the increases in solar radiation caused by opening of canopy leads to further drying and offers an opportunity for weed invasion. The severity and area burnt by wildfires is projected to continue to increase over the coming decades as climate change advances^69–71^, which has clear implications for *P. kundagungan* and *P. richmondensis*.

Forecast increases in temperature, rainfall variability, frequency and severity of drought and wildfire is likely to cause large range contractions for these narrowly distributed montane species beyond what has already been predicted in the present study, with no capacity for upslope range expansions. Ultimately, under the pressures of unmitigated climate change, amphibian species adapted to montane rainforest environments will to continue to decline to extinction.

### Management implications

Forecast increases in temperature, rainfall variability, frequency and severity of drought and wildfire is likely to cause large range contractions for these narrowly distributed montane species within two decades. All existing populations are isolated and characterised by a small number of individuals^46^ and as such they are already at risk of stochastic events resulting in localised population declines and extinctions^45^. A fundamental tenant of the declining-population paradigm is that the agent of decline must be alleviated for populations to persist^72^. However, it seems unlikely that the impacts of climate change will be abated in time for these species to persist beyond very small climatic refuges identified via our modelling by 2055. Despite this, we argue that there are measures that can be applied to conserve these species in the wild and increase population resilience.

Firstly, it is imperative that captive insurance populations be established immediately. It is currently unknown if these species can be maintained and bred in captivity and the development of husbandry protocols may take many years to establish. This should include collections of the full suite of genetic variability from across the species' range, with a view to undertaking conservation translocations (see Scheele et al.^73^). Translocations could include reintroductions to extirpated sites using targeted gene flow to select for desiccation tolerance^74^, population augmentation to bolster existing populations (e.g. head-starting) and assisted colonisation, where animals are released into areas outside of their existing range. The latter is perhaps the most contentious because it may involve movement of allopatric species into the range of sister taxa.

Secondly, although we have identified the distribution of climatic refuges for both target species, defined here as areas that offer climate refuge under a high-warming scenario in 2055, alleviating additional threats in these areas is vital to long-term conservation planning for climate change^75–77^. Although these climatic refuges are all protected, actions to alleviate additional threats, such as control of feral pigs (*Sus scrofa*), weed and fire management, should be prioritised in these areas to ensure habitat viability.

The impact of climate change on *P. kundagungan* and *P. richmondensis* is an ongoing process, and the predictions of this study should be tested to determine the species' actual response. Baseline data and monitoring methods exist for both species^45,46^, which will allow future studies to effectively monitor range contractions. Monitoring data should serve as the primary tool to assess the effectiveness of conservation actions.

Ultimately, under the pressures of unmitigated climate change, *P. kundagungan* and *P. richmondensis* along with many other amphibian species adapted to cool, moist montane rainforest environments face a challenging future. We argue that both short and long-term management pathways exist to conserve these species in the wild and increase population resilience. However, with large areas of habitat already impacted, conservation efforts for both these species need to be initiated urgently.

## Data Availability

Supporting data is available on request from Liam Bolitho, Faculty of Science and Engineering, Southern Cross University, PO Box 157, Lismore, New South Wales 2480, Australia.
